# Development of a chimeric Zika vaccine using a licensed live-attenuated flavivirus vaccine as backbone

**DOI:** 10.1038/s41467-018-02975-w

**Published:** 2018-02-14

**Authors:** Xiao-Feng Li, Hao-Long Dong, Hong-Jiang Wang, Xing-Yao Huang, Ye-Feng Qiu, Xue Ji, Qing Ye, Chunfeng Li, Yang Liu, Yong-Qiang Deng, Tao Jiang, Gong Cheng, Fu-Chun Zhang, Andrew D. Davidson, Ya-Jun Song, Pei-Yong Shi, Cheng-Feng Qin

**Affiliations:** 1 State Key Laboratory of Pathogen and Biosecurity, Beijing Institute of Microbiology and Epidemiology, Beijing, 100071 China; 2 Guangzhou Eighth People’s Hospital, Guangzhou Medical University, Guangzhou, 510060 China; 3 Department of Infection Control, The 306th Hospital of PLA, Beijing, 100101 China; 4 Laboratory Animal Center, Academy of Military Medical Science, Beijing, 100071 China; 5 Center for Systems Medicine, Institute of Basic Medical Sciences, Chinese Academy of Medical Sciences and Peking Union Medical College, Beijing, 100005 China; 6 Tsinghua-Peking Center for Life Sciences, School of Medicine, Tsinghua University, Beijing, 100084 China; 7 Faculty of Biomedical Sciences, School of Cellular and Molecular Medicine, University of Bristol, University Walk, Bristol, BS8 1TD UK; 8 Department of Biochemistry and Molecular Biology, Sealy Center for Structural Biology and Molecular Biophysics, University of Texas Medical Branch, Galveston, Texas 77555 USA; 9 Department of Pharmacology and Toxicology, University of Texas Medical Branch, Galveston, Texas 77555 USA

## Abstract

The global spread of Zika virus (ZIKV) and its unexpected association with congenital defects necessitates the rapid development of a safe and effective vaccine. Here we report the development and characterization of a recombinant chimeric ZIKV vaccine candidate (termed ChinZIKV) that expresses the prM-E proteins of ZIKV using the licensed Japanese encephalitis live-attenuated vaccine SA14-14-2 as the genetic backbone. ChinZIKV retains its replication activity and genetic stability in vitro, while exhibiting an attenuation phenotype in multiple animal models. Remarkably, immunization of mice and rhesus macaques with a single dose of ChinZIKV elicits robust and long-lasting immune responses, and confers complete protection against ZIKV challenge. Significantly, female mice immunized with ChinZIKV are protected against placental and fetal damage upon ZIKV challenge during pregnancy. Overall, our study provides an alternative vaccine platform in response to the ZIKV emergency, and the safety, immunogenicity, and protection profiles of ChinZIKV warrant further clinical development.

## Abstract (web summary)

Given the recent Zika virus (ZIKV) epidemic, development of an effective vaccine is of high importance. Here, the authors use a licensed live-attenuated flavivirus vaccine backbone to develop a ZIKV vaccine and determine immunogenicity, safety and protection profiles in different animal models.

## Introduction

Zika virus (ZIKV) is a mosquito-borne virus belonging to the *Flavivirus* genus of the *Flaviviridae* family, which includes other important human pathogens such as yellow fever virus (YFV), dengue virus (DENV), Japanese encephalitis virus (JEV), West Nile virus (WNV), and tick-borne encephalitis virus (TBEV). Since 2007, ZIKV has caused epidemics in Polynesia, in the South Pacific, and most recently in the United States. Increasing evidence supports a casual association between ZIKV infection, microcephaly, and other congenital malformations in infants, as well as Guillain–Barré syndrome in adults^1–3^. In addition, unlike most other flavivirus members, ZIKV has the potential for significant human-to-human transmission through sexual and vertical routes^3,4^. These unexpected and unique properties underscore the urgent requirement for the development of a safe and effective ZIKV vaccine. The existence of a number of successful flavivirus vaccines and multiple vaccine development platforms, has facilitated remarkable progress in the field of ZIKV vaccine development. Recently, a range of non-replicating ZIKV vaccine candidates including; DNA vaccines expressing the viral prM-E genes, purified inactivated vaccines, and messenger RNA vaccines have been developed and evaluated in animals with promising results^5–10^, and some have now entered clinical trials^11,12^.

The immune mechanisms that mediate protection against ZIKV infection are not fully understood. In comparison with non-replicating vaccines, live-attenuated vaccines are designed to mimic the natural life cycle of the wild-type virus and are capable of inducing long-lasting immune responses with low production cost. These characteristics make live-attenuated vaccines the most effective countermeasure against many viral diseases, as exemplified by the two flavivirus vaccines, YFV vaccine strain 17D (YFV 17D), and JE vaccine virus SA14-14-2 (JEV SA14-14-2). Both vaccines are recognized for their excellent safety and efficacy profiles in humans. Neutralizing antibodies targeting flavivirus structural proteins prevent or reduce viral replication, leading to the clearance of extracellular viruses and commonly correlate with protection from disease. For example, neutralizing antibody titers (> 10), which correlate with protective efficacy, have been reported for flavivirus vaccines including YFV, JEV, and TBEV^13–15^. CD8^+^ T cells limit the spread of viruses by either recognizing and killing infected cells or secreting specific antiviral cytokines, whereas CD4^+^ T cells contribute to protection through cytokine production and support the generation and maintenance of antibody and CD8^+^ T-cell responses. Multiple studies have suggested that the T-cell response is a critical contributor to protective immunity in flavivirus infections^16–18^. Recently, two live-attenuated ZIKV vaccine candidates have been generated by reverse genetics and shown capable of inducing sterilizing immunity in animal models^10,19,20^.

Flaviviruses have an 11 kb single-stranded positive-sense RNA genome that contains a single open reading frame (ORF) flanked by two untranslated regions (5′- and 3′-UTRs). The ORF encodes a single polyprotein, which is proteolytically processed into three structural proteins (C, prM, and E) and seven nonstructural proteins (NS1, NS2A, NS2B, NS3, NS4A, NS4B, and NS5). One platform for the development of flavivirus vaccine candidates has been the creation of a recombinant chimeric flavivirus in which the prM and E genes of a live-attenuated flavivirus vaccine strain are substituted with the corresponding genes of a heterologous flavivirus^21^. Recombinant live-attenuated chimeric DENV (CYD-TDV) and JEV vaccines, using YFV 17D as a backbone, have been well characterized in terms of immunogenicity and safety, and are now commercially available in several countries^22,23^. Several other chimeric vaccines based on different attenuated strains are currently under different stages of development^24,25^. The live-attenuated JEV vaccine virus SA14-14-2, recently prequalified by the World Health Organization (WHO), has been administered to more than 400 million children^26^. Large-scale vaccination programs in various countries have shown that the vaccine possesses an excellent safety profile and remarkable effectiveness and efficacy^27–29^. The phenotypic and genotypic characteristics that correlate with attenuation are highly stable^26^. These attractive features of JEV SA14-14-2 make it an ideal genetic backbone for the development of chimeric flavivirus vaccines^30^.

In the present study, we develop a chimeric live-attenuated ZIKV vaccine candidate, using JEV SA14-14-2 as a backbone, and through extensive in vitro and in vivo characterization demonstrate that the chimeric virus is a promising ZIKV vaccine candidate, which deserves further clinical development.

## Results

### Construction and characterization of ChinZIKV

**Fig. 1:**
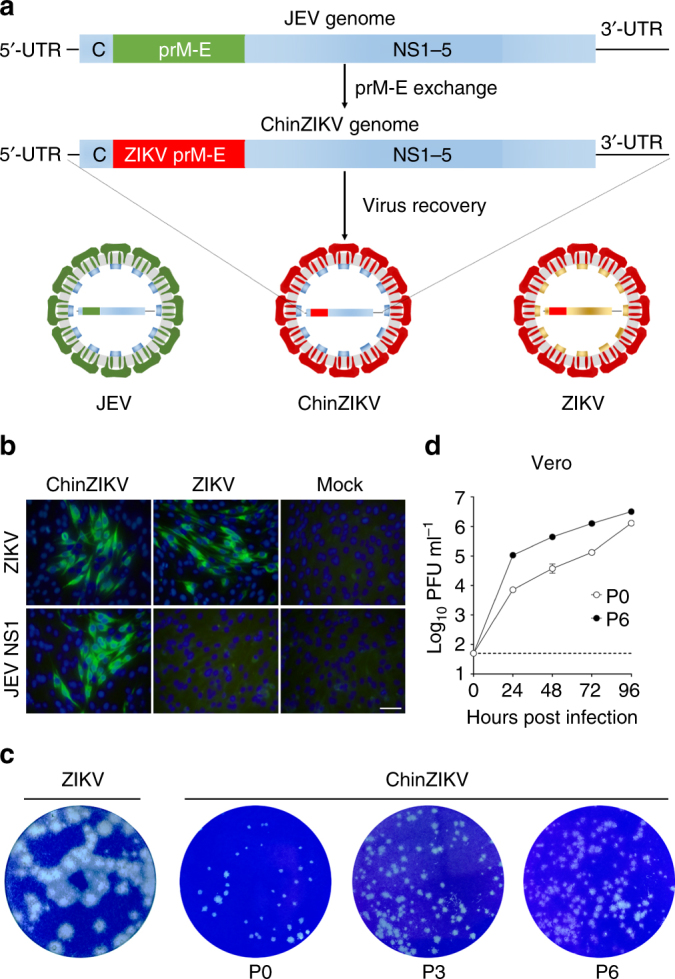
Construction and characterization of ChinZIKV. **a** Strategy for construction of the chimeric ZIKV vaccine ChinZIKV and schematic representation of its genome. **b** Immunostaining of BHK-21 cells infected with ChinZIKV-, ZIKV-, or mock-infected at an MOI of 0.01 at 48 h post-infection with ZIKV patient anti-serum and JEV NS1 antibodies. Scale bar: 100 μm. **c** Plaque morphology of ZIKV, the recovered ChinZIKV, and ChinZIKV at passages 3 and 6 on BHK-21 cells. BHK-21 cells were infected with the indicated viruses, and plaques were developed after 96 h. **d** Growth curves of the recovered ChinZIKV and ChinZIKV at passage 6 in Vero cells. Vero cells were infected with the indicated viruses at an MOI of 2 and the cell supernatants were collected at the indicated times for determination of virus titers by plaque assay using BHK-21 cells. The data are representative of at least three independent experiments and error bars indicate the SD

Using standard reverse genetics technology, we rationally designed and generated a JEV-ZIKV chimera (named ChinZIKV) by replacement of the prM-E genes of JEV SA14-14-2 with the corresponding region of an Asian ZIKV strain FSS13025^31^ (Fig. 1a). The resulting chimeric virus, ChinZIKV, was successfully constructed and rescued in BHK-21 cells and confirmed by full genome sequencing. Accordingly, the chimera expressed the structural proteins of ZIKV and the non-structural proteins of JEV as verified by immunostaining (Fig. 1b and Supplementary Fig. 1a) and western blotting (Supplementary Fig. 1b), respectively. ChinZIKV caused cytopathic effects (CPE) at low multiplicities of infection and exhibited a small-plaque phenotype in BHK-21 cells, which is a potential attenuation marker, compared with the parental ZIKV (Fig. 1c). Similar to other flaviviruses, ChinZIKV replicated efficiently in both mammalian and mosquito cells, with a peak titer of 10^6^ PFU ml^–1^ in Vero cells, which is a certified cell type for vaccine production by the WHO (Fig. 1d and Supplementary Fig. 1c).

Genetic stability is one of essential properties required of a potent live-attenuated vaccine. To test this, we serially passaged ChinZIKV in Vero cells six times. Full-genome sequence analysis of the 6th passaged virus (P6) revealed a total of six nucleotide mutations, resulting in single amino acid substitutions in the prM and NS1 proteins, which accumulated during the serial passages (Supplementary Table 1), possibly related to adaptation to Vero cells. Phenotypic comparison showed no apparent change in plaque morphology at different passages (Fig. 1c and Supplementary Fig. 1c) and the P6 virus showed more efficient replication in Vero cells compared with the P0 virus (Fig. 1d and Supplementary Fig. 1d).

### Characterization of the attenuation phenotype of ChinZIKV

**Fig. 2:**
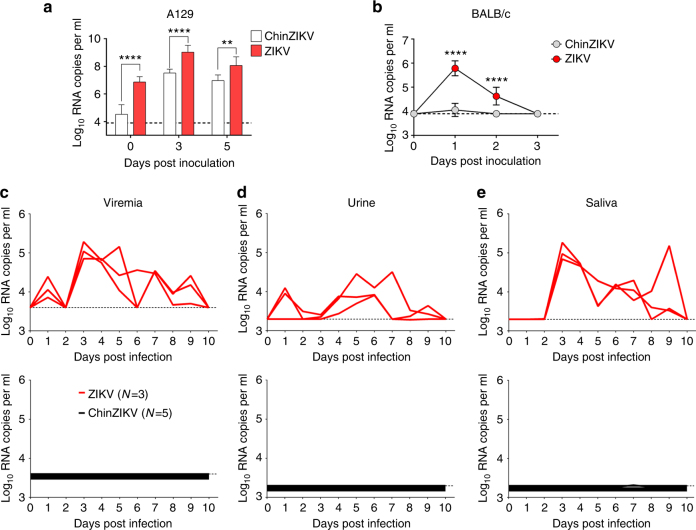
ChinZIKV is highly attenuated in mice and non-human primates. **a**, **b** Viral load in serum in mice inoculated with ChinZIKV or ZIKV. Four-week-old male mice were infected with 10^3^ (A129) or 10^5^ PFU (BALB/c) of the indicated viruses by the s.c. or i.p. routes, respectively. Viral RNA loads were determined by reverse transcription-quantitative PCR (RT-qPCR). Dotted lines indicate the detection limit. The data are representative of at least three independent experiments, and error bars indicate the SD. Significance was calculated using a two-way ANOVA with multiple comparison tests **a**, **b** (**P-value < 0.01; ****P-value < 0.0001). Viral load in serum **c**, urine **d**, and saliva **e** of monkeys infected with ChinZIKV or ZIKV. Monkeys were infected with 10^5^ PFU of ChinZIKV or ZIKV by the s.c. route. Blood and tested fluids were collected at the indicated times. Viral RNA amounts in the tested samples were determined by RT-qPCR

The in vitro properties of ChinZIKV encouraged us to test the attenuation phenotype in vivo. We first examined the viremia and tissue distribution of ChinZIKV in the well-established immunodeficient A129 mouse model^32^. Although no statistically significant difference was observed between the body weights of mice infected with ChinZIKV or ZIKV up to 15 days post infection (Supplementary Fig. 2c), ChinZIKV showed a significant attenuation phenotype in comparison with the wild-type ZIKV, with a much weaker viremia profile (Fig. 2a), as well as decreased viral loads in spleens and testis (Supplementary Fig. 2a, b). In addition, the A129 mice were not found to be persistently infected with ChinZIKV, as evidenced by undetectable levels of viral RNA in the organs of infected mice at day 15 post infection (Supplementary Fig. 2d). Then, we inoculated immunocompetent BALB/c mice with ChinZIKV and wild-type ZIKV by the intraperitoneal (i.p.) route^7,33^. Consistent with the results obtained using the A129 mice, there was no significant difference in the body weights of mice inoculated with ChinZIKV or ZIKV (Supplementary Fig. 3a). Remarkably, barely detectable viremia was observed in the ChinZIKV-infected mice, whereas ZIKV infection induced a rapid and transient viremia in mice (Fig. 2b). Viral RNA was barely detectable in the spleens and testis of ChinZIKV-inoculated mice (Supplementary Fig. 3b, c).

We also tested the neurovirulence of ChinZIKV in 1-day-old suckling BALB/c mice following intracerebral (i.c.) inoculation, in comparison with the same doses of wild type ZIKV and the two licensed live-attenuated vaccines YFV 17D and JEV SA14-14-2. As shown in Supplementary Fig. 4, all suckling mice succumbed to i.c. inoculation with wild-type ZIKV within 20 days, whereas no death or neurological signs were observed in all ChinZIKV-inoculated mice during the observation period. As expected, the two live-attenuated vaccine viruses were neurovirulent and all animals died between days 7–9 post inoculation with either JEV SA14-14-2 or YFV 17D (Supplementary Fig. 4). These findings demonstrate that ChinZIKV has a significantly less neurovirulent phenotype than ZIKV as well as the two licensed flavivirus vaccine viruses.

Ideally, a live-attenuated vaccine candidate should lose the capability to be transmitted by vector mosquitoes. To characterize the infectivity of ChinZIKV in *Aedes aegypti*, equal doses of ChinZIKV and wild-type ZIKV were microinjected into the thorax of mosquitoes, and viral replication assayed on day 6 post inoculation. As shown in Supplementary Fig. 5, high levels of ZIKV RNA were detected in all tested mosquitoes, whereas only trace amounts of viral RNA were detected in ChinZIKV-inoculated mosquitoes at the same time. These data suggest almost undetectable replication of ChinZIKV in *A. aegypti* mosquitoes.

A non-human primate model for ZIKV infection, capable of developing measurable viremia and neutralizing cellular immune responses, has been well established by us and other groups^34–36^ as an indicator for vaccine safety and efficacy in humans. Using our rhesus macaque model, we compared the replication of ChinZIKV with the ZIKV epidemic strain GZ01^35^. Monkeys (*n* = 3) infected subcutaneously (s.c.) with 10^5^ PFU of ZIKV strain GZ01, all developed sustained viremia within 10 days post infection, whereas monkeys (*n* = 5) infected with 10^5^ PFU of ChinZIKV failed to exhibit any detectable viremia (Fig. 2c). In addition, viral RNA was readily detected in the urine and saliva of ZIKV-infected monkeys, but not ChinZIKV-inoculated monkeys (Fig. 2d, e). Together, the attenuation phenotype of ChinZIKV in mice and non-human primates was comparable or superior to previous licensed live-attenuated YFV, JEV, or chimeric DENV vaccines^21,37,38^.

### ZIKV-specific immune responses induced by ChinZIKV in animals

Next, we investigated the immunogenicity profile of ChinZIKV in mice and non-human primates. ZIKV contains only one serotype^39^ and a neutralization antibody threshold of > 1 : 10 is considered protective against flavivirus infection^13,14^. A single s.c. immunization of adult BALB/c mice with ChinZIKV induced high levels of both ZIKV-specific IgG and neutralizing antibodies on day 28 post immunization (Supplementary Fig. 6). Importantly, anti-ZIKV neutralizing antibodies (Geometric mean titer (GMT) = 26) were detected in all ChinZIKV-immunized mice, even at day 210 post immunization (Supplementary Fig. 6).

**Fig. 3:**
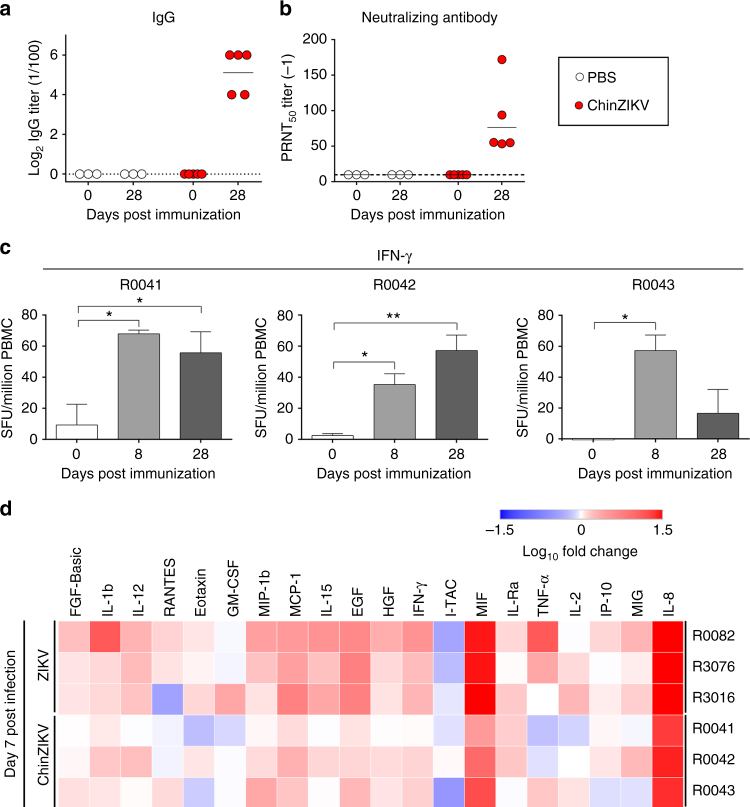
ChinZIKV elicits ZIKV-specific humoral and cellular immune responses in non-human primates. **a**, **b** Monkeys were immunized s.c. with a single dose of ChinZIKV (*n* = 5) or PBS as a control (*n* = 3). ZIKV-specific IgG and neutralizing antibody titers were determined by ELISA and PRNT_50_, respectively. Dotted lines indicate the limits of detection. **c** PBMCs from immunized monkeys were collected at the indicated times. The production of IFN-γ by PMBCs stimulated with the ZIKV E protein was measured by ELISPOT assay and expressed as spot-forming units (SFU) per 10^6^ PBMCs. Experiments were performed in duplicate (error bars represent SD). Significance was calculated using a one-way ANOVA with multiple comparison tests (*, *P*-value < 0.05; **, *P*-value < 0.01). **d** Heat map of the cytokine profile in sera from the monkeys inoculated with ChinZIKV or ZIKV at day 7 post inoculation. Each sample was analyzed using a Monkey Cytokine Magnetic 29-Plex Panel kit. Each cytokine level is summarized as the log_10_ of the ratio relative to baseline (Day 0 post infection)

Rhesus monkeys (*n* = 5) were then immunized with ChinZIKV by the s.c. route. High levels of ZIKV-specific IgG (GMT = 3,675) and neutralizing antibodies (GMT = 76) were detected on day 28 post immunization (Fig. 3a, b). No ZIKV-specific antibodies were detected in phosphate buffered solution (PBS)-immunized animals (*n* = 3), as expected. Although the precise role of the T-cell response during ZIKV infection remains largely unknown^40,41^, T-cell responses to the flavivirus E protein have been associated with protection in humans^42^. enzyme-linked immunosorbent spot (ELISPOT) assay showed that ZIKV E-specific interferon-γ (IFN-γ) secretion was significantly induced in peripheral blood mononuclear cells (PBMC) from ChinZIKV-immunized monkeys on day 8 post immunization (Fig. 3c). Other modest cellular immune responses, including the induction of interleukin (IL)-2 and IL-10, were also observed in ChinZIKV-immunized monkeys (Supplementary Fig. 7a, b), suggesting both Th1 and Th2 responses were elicited. In addition, Luminex bead assays showed a similar, but slightly weaker, pattern of cytokine induction in ChinZIKV-immunized monkeys compared with that found in natural ZIKV infection (Fig. 3d). Multiple cytokines with adaptive immunomodulatory roles, including IL-1b, IFN-γ, monocyte chemotactic protein 1 (MCP-1), macrophage migration inhibitory factor (MIF), macrophage inflammatory protein 1b (MIP-1b), IL-8, and IFN-gamma-inducible protein 10 (IP-10), were concurrently elevated upon either ZIKV infection or ChinZIKV immunization. These data demonstrate that ChinZIKV infection is capable of inducing humoral and cellular immune responses in mice and non-human primates that are similar to those raised by natural ZIKV infection.

### Efficacy of ChinZIKV in mice and non-human primates

**Fig. 4:**
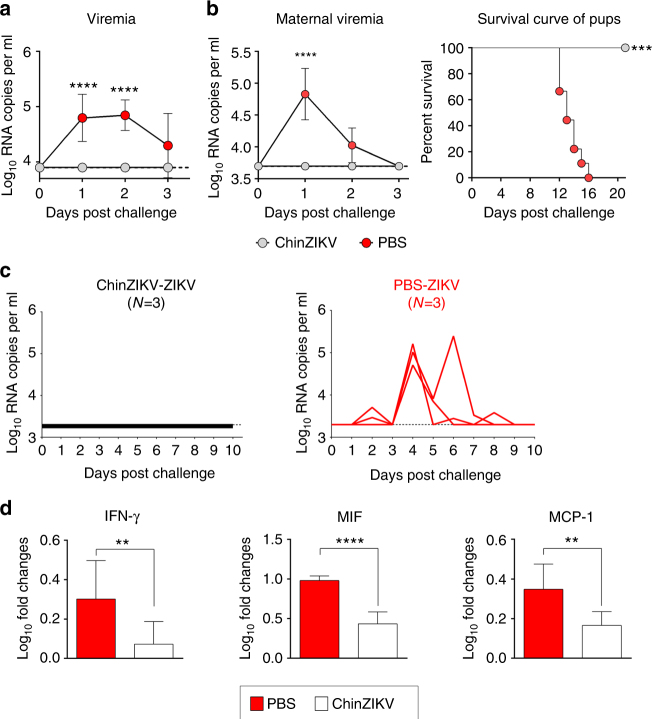
A single dose of ChinZIKV confers complete protection against ZIKV challenge in mice and monkeys. **a** Viral load in the sera of immunized mice after ZIKV challenge. The ChinZIKV immunized mice were challenged i.p. with 10^3^ PFU of ZIKV. Viral RNA loads were determined by RT-qPCR. Dotted lines indicate the detection limit. The data are representative of at least three independent experiments, and error bars indicate the SD. Significance was calculated using a two-way ANOVA with multiple comparison tests (****P-value < 0.0001). **b** Four-week-old female BALB/c mice were immunized with 10^4^ PFU of ChinZIKV (*n* = 3) or PBS (*n* = 2) as a control. On day 60 post immunization, the immunized mice were mated to 10-week-old male BALB/c mice. At embryonic day 13.5 (E13.5), the pregnant mice were infected with 10^5^ PFU of ZIKV by the i.p. route. Viral RNA loads at days 1–3 post-infection were determined by RT-qPCR. Dotted lines indicate the detection limit. The data are representative of at least three independent experiments, and error bars indicate the SD. Significance was calculated using a two-way ANOVA with multiple comparison tests (****P-value < 0.0001). One-day-old suckling mice born to the ChinZIKV-immunized (*n* = 15) or PBS-immunized (*n* = 9) dams were challenged i.c. with 100 PFU of ZIKV. The mice were then monitored for clinical symptoms and mortality for 21 days. Asterisks indicate values that are statistically significant (***P-value < 0.001). **c** The PBS- or ChinZIKV-immunized monkeys were challenged s.c. with 10^3^ PFU of ZIKV. Serum and body fluids were collected at the indicated times. Viral RNA amounts were determined by RT-qPCR. Dotted lines indicate the detection limit. **d** Cytokine amounts in the sera of the PBS- or ChinZIKV-immunized monkeys (*n* = 3, each group) at day 7 post challenge determined by a Monkey Cytokine Magnetic 29-Plex Panel kit. Experiments were performed in duplicate (error bars represent SD). Significance was calculated using the Student’s t test (**P-value < 0.01; ****P-value < 0.0001)

The induction of a robust neutralizing antibody response to ChinZIKV in animals led us to explore the protection afforded against challenge with a contemporary 2016 Venezuelan ZIKV strain^43^. We first examined the protective effect of ChinZIKV immunization using immunocompetent BALB/c mice. As expected, all BALB/c mice immunized with PBS developed a transient viremia upon s.c. challenge. Remarkably, none of the mice immunized with a single dose of ChinZIKV exhibited detectable viremia (Fig. 4a). We further tested whether ChinZIKV immunization protected pregnant mice and their offspring from ZIKV challenge. Supporting the previous experiment, viremia was not observed in the ChinZIKV-immunized pregnant mice following s.c. challenge with ZIKV (Fig. 4b). More importantly, the offspring born to ChinZIKV-immunized dams remained healthy after i.c. challenge with ZIKV and all littermates grew up without any neurological symptoms (Fig. 4b). In contrast, all offspring born to PBS-immunized dams died and had severe neurological symptoms, including hind limb weakness and paralysis, after i.c. challenge with ZIKV, as previously described (Fig. 4b)^44,45^. These results demonstrate that a single immunization with ChinZIKV confers protection of both pregnant dams and their offspring against wild type ZIKV challenge.

Furthermore, we tested the protective efficacy of ChinZIKV in a rhesus macaque monkey model. As shown in Fig. 4c, challenge of all PBS-immunized animals with wild-type ZIKV led to sustained viremia within 10 days post infection. Remarkably, monkeys immunized with a single dose of ChinZIKV were completely protected against ZIKV challenge, as evidenced by the lack of detectable viremia observed during the post-challenge period (Fig. 4c). In addition, Luminex bead assay showed that multiple cytokines were significantly elevated in the sera of PBS-immunized monkeys in comparison with ChinZIKV-immunized monkeys, upon ZIKV challenge (Supplementary Fig. 8a). Specifically, the levels of disease-related cytokines including IFN-γ, MIF, and MCP-1 were significantly lower in the sera of ChinZIKV-immunized monkeys compared with the control group (Fig. 4d). The levels of multiple cytokines including IL-2, IL-Ra, RANTES (regulated on activation, normal T cell expressed and secreted; CCL-5), and eotaxin showed a divergent profile between ChinZIKV-immunized and PBS-inoculated animals following ZIKV challenge (Supplementary Fig. 8b), which may contribute to the protection against flavivirus challenge^46^. The potent proinflammatory and T-cell responses, together with the production of high-titer-neutralizing antibodies, are likely to jointly contribute to the protection in the non-human primates.

### ChinZIKV prevents vertical transmission and fetus damage

**Fig. 5:**
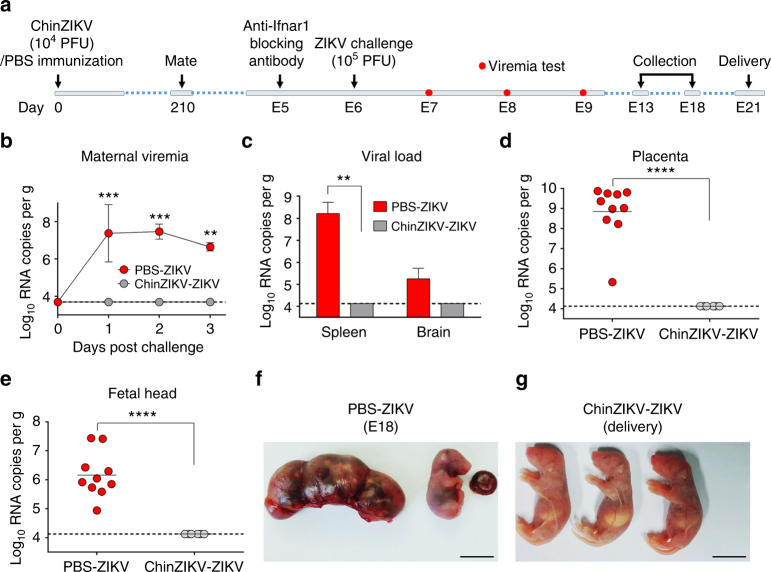
ChinZIKV immunization prevents vertical ZIKV transmission and fetus injury during pregnancy. **a** Scheme of immunization of BALB/c female mice with 10^4^ PFU of ChinZIKV or PBS. **b–e** At day 210 post immunization, vaccinated female mice were mated with BALB/c males. Pregnant mice (*n* = 3) were administered 2.0 mg of anti-Ifnar1-blocking antibody on E5 and 1 day later (E6) challenged with 10^5^ PFU of ZIKV. Viral RNA loads in serum at days 1–3 post challenge were determined by RT-qPCR **b**. On E13, animals were killed; maternal spleens and brains **c**, placenta **d**, and fetal heads **e** were collected and viral RNA levels were quantified by RT-qPCR. The data are representative of at least three independent experiments and error bars indicate the SD. **f**, **g** Outcome of fetuses from PBS or ChinZIKV vaccinated dams. Scale bar: 1 cm. **f** Representative images of hemorrhagic uterus (left) and moribund fetus (right) recovered from PBS-immunized moribund dams at E18. **g** Representative images of pups delivered at term to ChinZIKV vaccinated dams. Dotted lines indicate the detection limit. Significance was calculated using a two-way ANOVA with multiple comparison tests **b** (**P-value < 0.01; ***P-value < 0.001) or a Student’s *t*-test **c–e** (**P-value < 0.01; ****P-value < 0.0001)

Finally, to further test the ability of ChinZIKV immunization to prevent vertical ZIKV transmission during pregnancy^15,47^, we mated immunized BALB/c female mice with 10-week-old naive BALB/c male mice at day 210 post immunization. Pregnant dams were then administered 2.0 mg of anti-Ifnar1 blocking antibody at embryo day 5 (E5) and 1 day later (E6), challenged with 10^5^ PFU of ZIKV. Viral RNA loads in serum were determined at days 1–3 post challenge by RT-qPCR. At E13, animals were killed, and maternal tissues were collected and viral RNA levels quantified (Fig. 5a). As expected, following ZIKV challenge, ChinZIKV-immunized dams had no detectable viremia during the observation period, whereas PBS-immunized mice sustained high levels of viremia at the indicated time points (Fig. 5b). Consistent with this observation, high levels of viral RNA were detected in maternal spleens and brains in PBS-immunized mice, whereas no detectable viral RNA was observed in ChinZIKV-immunized mice (Fig. 5c). More importantly, viral RNA was not detectable in placentas and fetal heads from the ChinZIKV-immunized dams (Fig. 5d, e). In contrast, high levels of viral RNA were detected in the corresponding tissues from PBS-immunized dams (Fig. 5d, e). In addition, extensive placental injury and fetal demise were observed in PBS-immunized mice at E18, whereas all pups were delivered at term with normal viability from the ChinZIKV-immunized group (Fig. 5f, g). These results demonstrate that pre-conception maternal immunity induced by ChinZIKV immunization efficiently prevented vertical ZIKV transmission during pregnancy and protected the fetuses from ZIKV infection.

## Discussion

In the present study, we rationally engineered a chimeric ZIKV vaccine candidate by replacement of the prM-E genes of the commercial JE vaccine strain SA14-14-2 with the corresponding segment of ZIKV. As expected, the resulting chimeric ChinZIKV showed a significantly attenuated phenotype in multiple animal models. ChinZIKV is highly attenuated in mouse neurovirulence, even more strongly than the two commercial flavivirus live-attenuated vaccine strains JEV SA14-14-2 and YFV 17D (Supplementary Fig. 4). Recently, we have identified the major mouse neurovirulence determinant in the prM protein, which is closely linked to neurotropism and severe microcephaly in the fetus^48^. However, the chimeric ChinZIKV did not contain this virulence determinant, which further supports its safety profile. Significantly, immunocompetent mice and non-human primates inoculated s.c. with ChinZIKV did not exhibit detectable viremia (Fig. 2). This unique feature has been reported for other chimeric live-attenuated flaviviruses, including DENV, WNV, and TBEV, which have all been developed based on the JEV vaccine strain SA14-14-2^30,47,49^. We speculate that this difference to YFV-based chimeric vaccines may be attributed to the intrinsic characteristics of the JEV SA14-14-2 genetic backbone. Previous investigations have demonstrated that the JEV vaccine SA14-14-2 or other live-attenuated JEV vaccine candidates did not cause detectable viremia in mice or monkeys^50,51^. The underlying mechanisms accounting for the absence of vaccine viremia remain elusive and warrant further investigation.

Remarkably, immunization of mice and non-human primates with a single dose of ChinZIKV elicited a rapid and robust antibody response, conferring complete protection upon challenge with an epidemic ZIKV strain. Several non-replicating ZIKV vaccine candidates have been shown to induce high titers of neutralizing antibodies that contribute to the solid protective immunity in animals^7–9,52^. It is noteworthy that ChinZIKV seems to induce a higher level of neutralizing antibodies than an equal dose of wild type ZIKV in our monkey model (GMT of 76 for ChinZIKV on day 28 p.i. vs GMT of 30 for ZIKV on day 21 p.i.)^35^, which is probably associated with the higher replication rate of the JEV backbone. Recently, a vertical ZIKV transmission model has been used to evaluate the protective efficacy of ZIKV vaccine candidates^8^. In our study, a single dose of ChinZIKV can induce high levels of neutralizing antibodies against ZIKV and provide a complete prevention of vertical transmission to the fetus during pregnancy, even as late as day 210 post immunization. Similarly, several other vaccine candidates under development also prevented vertical transmission of ZIKV across the placental barrier, thus limiting fetal infection^10,19^. Collectively, these results from animal models suggest pre-conception maternal immunity is sufficient to protect against fetal transmission.

Recent data have demonstrated that the cellular immune response, especially CD8^+^ T cells, also contributes to protection against ZIKV infection^40,53^. Our study showed that ChinZIKV inoculation was able to elicit obvious secretion of IFN-γ from PMBCs isolated from monkeys upon stimulation with the ZIKV E protein. Both ChinZIKV and ZIKV infections also resulted in similar patterns of cytokine induction, with a slight weaker induction for ChinZIKV. Notably, the increased secretion of the chemokines MCP-1, involved in monocyte infiltration and IP-10, involved in the recruitment of activated effector T cells were observed in both groups. Notably, increased levels of IP-10 and MCP-1 have been found in ZIKV-infected monkeys and pregnant women with neonatal microcephaly^54–56^. These findings demonstrate the ChinZIKV chimera is able to induce a similar cellular immune response profile to that of ZIKV. Interestingly, following ZIKV challenge, the levels of IFN-γ, MIF, and MCP-1 were significantly lower in the ChinZIKV-immunized animals than that in the PBS control group. MIF has a broad range of immune and pro-inflammatory activities, and has been reported to be correlated with the severity of dengue disease^57,58^. MCP-1 is a potent chemotactic factor for monocytes and macrophages, and higher MCP-1 levels in placebo recipients than CYD-TDV recipients have been reported^59^. Reduction in the levels of these disease related cytokines indicates that immunization with the chimera confers a strong protective immune response against ZIKV infection in monkeys. On the other hand, the induction of a number of cytokines, including IL-2, IL-Ra, RANTES (CCL-5), and eotaxin diverged markedly between the ChinZIKV-immunized and PBS-inoculated animals after ZIKV challenge. It has been reported that chemokine receptor CCR5 functioned as a key factor in viral clearance and survival in a mouse model of WNV encephalitis and WNV infection in humans^60,61^. Increased production of CCR5 in immunized-monkeys following ZIKV challenge might contribute to recovery from ZIKV infection.

Although several vaccine candidates have been trialed clinically^11^, the complexity of ZIKV immunity and pathogenesis has introduced uncertainty to ZIKV vaccine development^62,63^. In particular, recent reports that DENV antibodies can either neutralize or enhance ZIKV infection^8^ have brought additional levels of complexity to efforts to develop a safe and effective ZIKV vaccine. Live-attenuated vaccines, including the chimeric vaccine described here, that mimic the kind of protective immunity induced in people who survive natural ZIKV infection could be a rational approach to success. A recombinant live-attenuated ZIKV vaccine candidate has been generated by deletion of 10-nts in the viral 3′-UTR by reverse genetics, and a single dose immunization of mice and non-human primates conferred full protection against ZIKV challenge^10,20^. The recombinant ChinZIKV developed here is based on a licensed human vaccine, and can be easily and economically produced on a large-scale using a similar manufacturing and quality control system, providing a practical solution for resource-limited areas where ZIKV is endemic. However, considerable work is still needed to better define the biology of ZIKV in humans and in the design and analysis of clinical trials.

Overall, our preclinical results described here illustrate a safe, affordable, and effective recombinant chimeric ZIKV vaccine that deserves further clinical development in the near future. Our data on the cytokine production patterns following virus immunization and challenge represent valuable resources for further study of the mechanisms underlying protective immunity conferred by ChinZIKV, as well as other versions of live-attenuated vaccine candidates against ZIKV.

## Methods

### Cells and viruses

BHK-21 (baby hamster kidney, ATCC CCL-10) and Vero (African green monkey kidney, ATCC CCL-81) cells were maintained in Dulbecco’s minimal essential medium (DMEM; Thermo Fisher Scientific, USA) supplemented with 10% fetal bovine serum (FBS) and penicillin (100 U ml^–1^)–streptomycin (100 μg ml^–1^). *Aedes albopictus* C6/36 cells were cultured in RPMI 1640 supplemented with non-essential amino acids (Thermo Fisher Scientific) and 10% FBS. BHK-21 and Vero cell lines were grown at 37 °C in 5% CO_2_, whereas C6/36 cells were maintained at 28 °C. ZIKV strain GZ01 (GenBank accession no. KU820898) was originally isolated from the urine sample of a ZIKV patient returning from Venezuela to China in 2016^43^. ZIKV strain FSS13025 (GenBank accession number KU955593) was originally isolated from Cambodia in 2010 and recovered from an infectious clone of ZIKV^31^. The JEV vaccine strain SA14-14-2 and YFV vaccine strain 17D were from the Chengdu Institute of Biological Products and Beijing Institute of Biological Products, respectively. Virus titers were determined by standard plaque assay on BHK-21 cells, and virus stocks were stored in aliquots at − 80 °C until use.

### Genetic construction of the JEV/ZIKV chimera

All plasmids were constructed using standard molecular biology protocols and confirmed by enzyme digestion and DNA sequencing. Genetic construction of the full-length infectious clone of JEV has been previously described^30,49,64^. Cloning sites were engineered to permit replacement of the entire prM and E coding sequences of JEV with the corresponding sequences of the ZIKV strain FSS13025. The resulting plasmid contained the full-length complementary DNA of the JEV/ZIKV chimera (named pChinZIKV). Further details about the construction of pChinZIKV are available from the authors on request and the corresponding primers used in this study are listed in Supplementary Table 2.

### Transcription and transfection

The plasmid pChinZIKV was linearized with *Xho*I and used as a template for SP6 RNA polymerase transcription in the presence of an m^7^GpppA cap analog. In vitro transcription was done using the RiboMAX Large Scale Production System (Promega, USA) according to the manufacturer’s protocol. The yield and integrity of RNA transcripts was analyzed by gel electrophoresis under non-denaturing conditions. RNA transcripts (5 μg) were transfected with Lipofectamine 3000 (Thermo Fisher Scientific) into BHK-21 cells grown in 60 mm-diameter culture dishes. Supernatants were then collected at day 4 post transfection, when typical CPE were observed. Virus titers were determined by standard plaque assay on BHK-21 cells. Briefly, BHK-21 cells in 12-well plates were infected with a 10-fold serial dilution of viruses. The plates were incubated at 37 °C for 1 h and cells were overlaid with 1% low-melting point agarose (Promega) in DMEM containing 2% FBS. After further incubation at 37 °C for 4 days, the cells were fixed with 4% formaldehyde and stained with 0.2% crystal violet to visualize the plaques.

### Nucleotide sequencing

Viral RNA was isolated using a PureLink RNA minikit (Thermo Fisher Scientific) and genomic cDNA was obtained by RT using SuperScript III (Thermo Fisher Scientific). For determination of viral consensus sequences, PCR products were directly sequenced in both directions using virus-specific primers. Sequence fragments were assembled into a consensus sequence with DNA STAR software, version 7.0.

### Growth curves

Growth curves of ChinZIKV in BHK-21, C6/36, or Vero cells were performed in a 12-well plate at a multiplicity of infection (MOI) of 0.01 or 2. Cell supernatants were collected at successive 12 or 24 h intervals post infection. Viral titers were then quantitated by plaque assay on BHK-21 cells or by RT-qPCR as described below.

### Immunostaining assay

Immunostaining of ZIKV-infected cells were performed as previously described^43^. Briefly, confluent BHK-21 cells grown in six-well plates containing a 1 cm^2^ coverslip were infected with viruses at an MOI of 0.01. At 48 h post infection, the coverslips containing infected cells were removed and directly used for subsequent analysis. The cells on the coverslip were fixed with ice-cold acetone and incubated with convalescent sera from a ZIKV patient^43^ (1 : 200 dilution), a mouse monoclonal antibody against the E protein of ZIKV (1 : 1,000, Cat#BF-1176-56, BioFront Technologies, USA) or a mouse monoclonal antibody against the JEV NS1 protein (1 : 20, Cat#ab41651, Abcam, UK) at 37 °C for 1 h. The use of human sera was approved by the Institutional Review Board of Guangzhou Eighth People’s Hospital with written informed consent form the patient. Cells were washed three times with PBS and then incubated with secondary antibodies conjugated to Alexa Fluor 488 (anti-mouse IgG, 1 : 2,000, Cat#A-11059; anti-human IgG, 1 : 500, Cat#A-11059, Thermo Fisher Scientific) in PBS for 30 min at 37 °C. Fluorescent cells were examined using a fluorescence microscope (Olympus, Japan).

### Western blotting assay

Confluent C6/36 cells grown in a six-well plate were infected with viruses at an MOI of 0.1 or mock infected as control. At 72 h post infection, cell lysates were mixed with loading buffer (0.1 M Tris-HCl (pH 8.8), 20% glycerol, 1% dithiothreitol, and 3% SDS and 0.0025% bromophenol blue), heated for 5 min at 100 °C, cooled on ice, and run on a precast 12% SDS-polyacrylamide gel electrophoresisgel (Biorad, USA). Protein was transferred to polyvinylidene difluoride membranes and the membranes were blocked overnight at 4 °C in PBST (Dulbecco’s PBS + 0.2% V/V Tween 20 + 1% bovine serum albumin). Following overnight blocking, the membranes were incubated for 1 h at room temperature with PBST containing a mouse anti-ZIKV E antibody (1 : 1,000, Cat#BF-1176-56, BioFront Technologies). Membranes were then washed three times with PBST and incubated for 1 h at RT with PBST containing a 1 : 1,000 dilution of sheep anti-mouse horseradish peroxidase (HRP). Membranes were then washed three times with PBST and developed using a Pierce ECL western blotting substrate (Cat#32109, Thermo Fisher Scientific) according to the manufacturer’s recommendation.

### Genetic stability assay

The recovered virus ChinZIKV was consecutively passaged in Vero cells for six passages. The complete genome sequence, growth curve, and plaque phenotype of the passaged viruses (P3 or P6) were assessed as described above. The sixth passage virus (P6) was titered and prepared as a stock for further in vivo studies.

### Enzyme-linked immunosorbent assay

Enzyme-linked immunosorbent assay (ELISA) was used to determine the IgG antibody titers in the sera of animals. Briefly, 96-well plates were coated overnight at 4 °C with 1 mg ml^–1^ of recombinant ZIKV E protein^65^ in a pH 9.6 carbonate buffer. Subsequently, the coated plates were incubated with serial dilutions of serum collected from the immunized animals. After incubation with peroxidase-conjugated goat anti-mouse IgG (1 : 5,000) (KPL, USA) for 1 h, the plates were then incubated with 3,3’,5,5’-tetramehylbenzidine substrate (Promega). The reaction was stopped by the addition of 2 N H_2_SO_4_ to the medium and the absorbance (450 nm) was read using a microplate reader (Beckman, USA). ELISA endpoint titers were defined as the highest reciprocal serum dilution that yielded an absorbance > 2-fold over background values.

### Neutralization assay

Neutralizing antibody titers were determined by a standard 50% plaque reduction neutralization test (PRNT_50_). Briefly, a 1 : 10 dilution of serum was prepared in DMEM containing 2% FBS and then heat inactivated for 30 min at 56 °C. Serial two- or fourfold dilutions of inactivated serum were mixed with equal volumes of viral solution to yield a mixture containing ~ 250 PFU of virus per ml. After incubation at 37 °C for 1 h, 200 μl volumes of the virus–antibody mixtures were added to wells of 12-well plates containing confluent monolayers of BHK-21 cells. Viral titers were then determined using the plaque assay described above. The endpoint neutralization titer was calculated by the method of Spearman–Karber^66^.

### Enzyme-linked immunosorbent spot

Cellular immune responses elicited from PBMCs of monkeys were assessed using the IFN-γ, IL-2 or IL-10 ELISPOT human set (Abcam) according to the manufacturer’s protocol. Briefly, PBMCs collected from the immunized or challenged rhesus monkeys were thawed and washed with Hanks balanced salt solution (HBSS). The cells were then centrifuged at room temperature at 2,500 r.p.m. for 15 min without braking, followed by two washes with HBSS. Then, the cells were re-suspended in RPMI 1640 containing 10% FBS and diluted to a working concentration of 5 × 10^6^ cells per ml. For ELISPOT analysis, 0.5 or 0.1 million cells per well were seeded and stimulated with recombiant ZIKV E protein prepared in our lab^65^ in 96-well tissue culture dishes coated with 5 mg ml^–1^ of IFN-γ, IL-2, or IL-10 capture monoclonal antibody. Non-stimulated and PMA (Merck, Germany)-stimulated cells were used as negative and positive controls, respectively. The cells were then cultured for 20 h at 37 °C and 5% CO_2_. Plates were washed and biotinylated anti-human IFN-γ, IL-2, or IL-10 antibody was added to each well and incubated for 2 h at room temperature. Thereafter, the plates were washed and incubated for 1 h at room temperature with streptavidin–HRP. Finally, AEC substrate solution (Abcam) was added and spots were counted with an ELISPOT Analysis System (At-Spot-2100, China). Assay results are expressed as the value obtained by the following: (number of spots in experimental well-number of spots in medium control)/10^6^ cells.

### Ethical statement

Research involving animals was approved by and carried out in strict accordance with the guidelines of the Institutional Experimental Animal Welfare and Ethics Committee (IACUC-13-2016-001).

### Mouse studies

BALB/c and A129 mice used in this study were purchased from Animal Laboratory Animal Center, Academy of Military Medical Science.

For attenuation tests, groups of 4-week-old male BALB/c or A129 mice were inoculated by the s.c. or i.p. route with the indicated doses of ChinZIKV or ZIKV, respectively. The body weight of infected mice was monitored for 15 days. Blood was collected at days 1–3 (BALB/c mice) or 1, 3, and 5 (A129 mice) post infection for determination of viral RNA in sera by RT-qPCR. Testis and spleens were collected at day 3 or day 7 post infection, to detect viral load by RT-qPCR.

For neurovirulence tests, 1-day-old suckling BALB/c mice were inoculated i.c. with 10 PFU of ChinZIKV, YFV 17D, or JEV SA14-14-2, respectively. Animals were monitored for 21 days after inoculation. Virus doses inducing a 50% mortality rate were calculated using the method of Reed and Muench^67^.

To test whether persistent infection of ChinZIKV occurred in mice, 6-week-old male A129 mice were infected s.c. with 10^3^ PFU of the chimera. Multiple organs were collected for viral RNA detection on days 6 and 15 post infection.

Immunogenicity was assessed by inoculation s.c. with 10^4^ PFU of ChinZIKV diluted in DMEM plus 2% FBS into 4-week-old female BALB/c mice. The mice were bled by tail vein puncture 1 day before (Day 0) and at 4 weeks post immunization. The sera were stored at − 20 °C for the determination of IgG by ELISA and neutralizing antibodies by PRNT_50_. For the efficacy assay, immunized mice were challenged i.p. with 10^3^ PFU of ZIKV strain GZ01 at 4 weeks post immunization. Blood and testis were collected as described above for determination of viral load in sera or the tested tissues, respectively.

For viral protection tests in pregnant mice and their offspring, 4-week-old female BALB/c mice were immunized with 10^4^ PFU of ChinZIKV or PBS. On day 60 post immunization, the immunized mice were mated to 10-week-old male BALB/c mice. At embryonic day 13.5 (E13.5), the pregnant mice were infected with 10^5^ PFU of ZIKV by the i.p. route. Viral RNA loads at days 1–3 post infection were determined by RT-qPCR. One-day-old suckling mice born to immunized dams were challenged i.c. with 100 PFU of ZIKV. The mice were then monitored for clinical symptoms and mortality for 21 days.

The vertical transmission model of ZIKV was recently described^15,47^. Briefly, 4-week-old female BALB/c mice were inoculated s.c. with 10^4^ PFU of ChinZIKV or PBS. On day 210 post immunization, immunized female mice were mated with naive 10-week-old male BALB/c mice. At E5, pregnant dams were injected i.p. with 2 mg of mouse anti-Ifnar1 antibody (clone MAR1-5A3; Leinco Technologies, USA). On E6, mice were challenged s.c. with 10^5^ PFU of ZIKV. Viral RNA loads in serum at days 1–3 post-challenge were determined by RT-qPCR. A set of animals were killed on E13 and analyzed for viral loads in placentas, fetuses, and maternal tissues as indicated. One of the PBS-immunized animals was killed on E18 to test the outcome of fetuses. One of the ChinZIKV-immunized dams was observed until delivery of pups at term.

### Monkey experiments

All experimental procedures involving rhesus monkeys were performed under sodium pentobarbital anesthesia by trained technicians and all efforts were made to ameliorate the welfare and to minimize animal suffering in accordance with the recommendations in The Use of Non-Human Primates in Research.

For immunization and challenge of rhesus monkeys, a total of 11 adult rhesus monkeys (weighing 4.5–6.0 kg) in good health were prescreened as negative for IgG antibodies against flaviviruses (including ZIKV, DENV, JEV, and YFV) by ELISA assay. Detailed information is provided in Supplementary Table 3. All the animals were housed individually in a single cage in a pathogen-free facility and acclimatized for a week. Rhesus monkeys were inoculated s.c. with 0.5 ml of viral solution containing 10^5^ PFU (*n* = 5) of ChinZIKV or ZIKV (*n* = 3), or were sham inoculated with PBS (*n* = 3). After the inoculation, clinical signs were recorded during a 10-day observation period. Blood and major body fluids were collected for the determination of viral load. Blood was collected daily for 10 days to detect viremia. Blood samples for neutralizing-antibody tests were taken before immunization (day 0) and then on day 28 post immunization. On day 55 post immunization, three of the animals were immunized with 10^5^ PFU of ChinZIKV and the PBS controls challenged by inoculation s.c. with 10^3^ PFU of the ZIKV strain GZ01. For the following 10 days, blood was collected for the determination of viremia. Neutralizing antibody levels in serum were measured by PRNT_50_ as described above.

Urine was collected from a container under the animal’s cage and was stored at − 80 °C until analysis. Saliva was obtained by running a sterile swab under the animal’s tongue. Swabs were placed immediately into 1.0 ml of viral transport medium (tissue culture medium 199 supplemented with 0.5% FBS and 1% antibiotic/antimycotic) for 60 min. Samples were vortexed vigorously, then centrifuged for 10 min at 1,000 × *g* before removing the swabs. Samples were stored at − 80 °C until processing.

Viral RNA was extracted from 0.1 ml of serum or 0.2 ml of urine and saliva samples using a PureLink RNA minikit (Thermo Fisher Scientific) according to the manufacturer’s instructions. RNA was eluted in 60 μl of RNase-free water, aliquoted, and stored at − 80 °C until use.

### Thoracic inoculation of mosquitoes

Detailed procedures for microinjection in mosquitoes have been described previously^68^. In brief, female *A. aegypti* mosquitoes (Rockefeller strain) were anesthetized on a cold tray, and subsequently a certain titre (0.2 PFU) of ZIKV or ChinZIKV was microinjected into the mosquito thoraxes.

### Viral RNA quantification in animal serum and tissues by RT-qPCR

The determination of viral RNA amounts by RT-qPCR was performed as described previously^35,69^. Briefly, the *Xho*I-linearized plasmids containing the full-length genomic cDNA clones of ChinZIKV or ZIKV were subjected to in vitro transcription using the RiboMAX Large Scale RNA Production System (Promega). The resulting RNA transcripts were purified using a PureLink RNA minikit (Thermo Fisher Scientific) according to the manufacturer’s instructions and quantified using spectrophotometry on Nanodrop 2000. The purified RNA was diluted 10-fold serially using RNase-free water and was detected using RT-qPCR. Threshold cycle (Ct) values for the known concentrations of the RNA were plotted against the log of the number of genome equivalent copies. The resultant standard curve was used to determine the number of genome equivalents of viral RNA in samples. The determination of the detection limit was based on the lowest level at which viral RNA was detected and remained within the range of linearity of a standard curve (Ct value of 38.5). RT-qPCR was performed using One Step PrimeScript RT-PCR Kit (Takara, Japan) with the primers and probes described in Supplementary Table 2. The 20 μl reaction mixtures were set up with 2 μl of RNA. Cycling conditions were as follows: 42 °C for 5 min, 95 °C for 10 s, followed by 40 cycles of 95 °C for 5 s and 60 °C for 20 s.

### Determination of viral load in mosquitoes by RT-qPCR

Total RNA was isolated from homogenized mosquitoes using an RNeasy Mini Kit (Qiagen, Germany) and reverse-transcribed into cDNA using an iScript cDNA synthesis kit (Bio-Rad, USA). Viral genomes were quantified via RT-qPCR amplification of ZIKV genes, normalized against *A. aegypti* actin (AAEL011197). The detection limit of ZIKV E/Actin mRNA ratio is 0.001. The primers and probes used for this analysis are shown in Supplementary Table 2.

### Cytokine secretion assays

Plasma samples of infected monkeys collected at days 0 and 7 post infection were stored frozen until analysis for cytokine production. Plasma concentrations of epidermal growth factor, eotaxin, fibroblast growth factor-basic, G-GSF, granulocyte–macrophage colony-stimulating factor, hepatocyte growth factor, IFN-γ, IL-1β, IL-1RA, IL-2, 4, 5, 6, 8, 10, 12, 15, 17, IP-10, I-TAC, MCP-1, MDC, MIF, MIG, MIP-1α, MIP-1β, RANTES, tumor necrosis factor-α, and vascular endothelial growth factor were measured with a Monkey Cytokine Magnetic 29-Plex Panel kit (Thermo Fisher Scientific) according to the manufacturer’s instruction. Antibody-coated magnetic microspheres optimized for quantifying non-human primate specific cytokines were premixed and the multiplexed assay was performed according the manufacturer’s instructions. Cytokine capture was performed overnight at 4 °C. Biotinylated cytokine detection antibodies and streptavidin–phycoerythrin (Thermo Fisher Scientific) were used at the manufacturer’s recommended dilution. Assays were performed on a Luminex 200™ machine equipped with Bio-Plex Manager Software (version 5.0) (Bio-Rad). The heatmap was produced using a web-based tool Morpheus and the original data were provided in Supplementary Tables 4 and 5.

### Statistical analysis

For viral burden analysis, an unpaired two-tailed *t* test was used to determine statistically significant differences. For three groups, variation between groups was measured by two-way analysis of variance with a multiple comparison testing. For survival analysis, Kaplan–Meier survival curves were analyzed by a log rank test. Statistical analyses were performed by using standard GraphPad Prism software, version 5.0.

### Data availability

The authors declare that all relevant data are available from the corresponding author upon request.

## Electronic supplementary material

Supplementary Information

## References

[1] Cao-Lormeau VM (2016). Guillain-Barré syndrome outbreak associated with zika virus infection in french polynesia: a case-control study. Lancet.

[2] Driggers RW (2016). Zika virus infection with prolonged maternal viremia and fetal brain abnormalities. N. Engl. J. Med..

[3] Mlakar J (2016). Zika virus associated with microcephaly. N. Engl. J. Med..

[4] D’Ortenzio E (2016). Evidence of sexual transmission of zika virus. N. Engl. J. Med..

[5] Richner JM (2017). Modified mRNA vaccines protect against zika virus infection. Cell.

[6] Griffin BD (2017). DNA vaccination protects mice against zika virus-induced damage to the testes. Nat. Commun..

[7] Larocca RA (2016). Vaccine protection against zika virus from brazil. Nature.

[8] Abbink P (2016). Protective efficacy of multiple vaccine platforms against zika virus challenge in rhesus monkeys. Science.

[9] Dowd KA (2016). Rapid development of a DNA vaccine for zika virus. Science.

[10] Shan C (2017). A single-dose live-attenuated vaccine prevents zika virus pregnancy transmission and testis damage. Nat. Commun..

[11] Gaudinski, M. R. et al. Safety, tolerability, and immunogenicity of two Zika virus DNA vaccine candidates in healthy adults: randomised, open-label, phase 1 clinical trials. *Lancet*10.1016/S0140-6736(17)33105-7 (2017). 10.1016/S0140-6736(17)33105-7 PMC6379903 29217376

[12] Tebas, P. et al. Safety and immunogenicity of an anti-Zika virus DNA vaccine - preliminary report. *N. Engl. J. Med.*10.1056/NEJMoa1708120 (2017). 10.1056/NEJMoa1708120 PMC6824915 34525286

[13] Hombach J, Solomon T, Kurane I, Jacobson J, Wood D (2005). Report on a WHO consultation on immunological endpoints for evaluation of new Japanese encephalitis vaccines, WHO, Geneva, 2–3 September, 2004. Vaccine.

[14] Kreil TR, Burger I, Bachmann M, Fraiss S, Eibl MM (1997). Antibodies protect mice against challenge with tick-borne encephalitis virus (TBEV)-infected macrophages. Clin. Exp. Immunol..

[15] Mason RA, Tauraso NM, Spertzel RO, Ginn RK (1973). Yellow fever vaccine: direct challenge of monkeys given graded doses of 17D vaccine. Appl. Microbiol..

[16] Turtle L (2016). Human T cell responses to Japanese encephalitis virus in health and disease. J. Exp. Med..

[17] Watson AM, Lam LK, Klimstra WB, Ryman KD (2016). The 17D-204 vaccine strain-induced protection against virulent yellow fever virus is mediated by humoral immunity and CD4+but not CD8+T cells. PLoS Pathog..

[18] Yauch LE (2010). CD4+T cells are not required for the induction of dengue virus-specific CD8+T cell or antibody responses but contribute to protection after vaccination. J. Immunol..

[19] Richner JM (2017). Vaccine mediated protection against zika virus-induced congenital disease. Cell.

[20] Shan C (2017). A live-attenuated Zika virus vaccine candidate induces sterilizing immunity in mouse models. Nat. Med..

[21] Monath TP (2015). Live virus vaccines based on a yellow fever vaccine backbone: standardized template with key considerations for a risk/benefit assessment. Vaccine.

[22] Chokephaibulkit K, Houillon G, Feroldi E, Bouckenooghe A (2016). Safety and immunogenicity of a live attenuated Japanese encephalitis chimeric virus vaccine (IMOJEV(R)) in children. Expert. Rev. Vaccin..

[23] Guy B, Jackson N (2016). Dengue vaccine: hypotheses to understand CYD-TDV-induced protection. Nat. Rev. Microbiol..

[24] Osorio JE, Wallace D, Stinchcomb DT (2016). A recombinant, chimeric tetravalent dengue vaccine candidate based on a dengue virus serotype 2 backbone. Expert. Rev. Vaccin..

[25] Whitehead SS (2016). Development of TV003/TV005, a single dose, highly immunogenic live attenuated dengue vaccine; what makes this vaccine different from the Sanofi-Pasteur CYD vaccine?. Expert Rev. Vaccines.

[26] Yu Y (2010). Phenotypic and genotypic characteristics of Japanese encephalitis attenuated live vaccine virus SA14-14-2 and their stabilities. Vaccine.

[27] Bista MB (2001). Efficacy of single-dose SA 14-14-2 vaccine against Japanese encephalitis: a case control study. Lancet.

[28] Hennessy S (1996). Effectiveness of live-attenuated Japanese encephalitis vaccine (SA14-14-2): a case-control study. Lancet.

[29] Kumar R, Tripathi P, Rizvi A (2009). Effectiveness of one dose of SA 14-14-2 vaccine against Japanese encephalitis. N. Engl. J. Med..

[30] Li XF (2013). A chimeric dengue virus vaccine using japanese encephalitis virus vaccine strain SA14-14-2 as backbone is immunogenic and protective against either parental virus in mice and nonhuman primates. J. Virol..

[31] Shan C (2016). An infectious cDNA clone of zika virus to study viral virulence, mosquito transmission, and antiviral inhibitors. Cell Host Microbe.

[32] Lazear HM (2016). A mouse model of zika virus pathogenesis. Cell Host Microbe.

[33] Zhang NN (2016). Characterization of the contemporary Zika virus in immunocompetent mice. Hum. Vaccin. Immunother..

[34] Dudley DM (2016). A rhesus macaque model of Asian-lineage Zika virus infection. Nat. Commun..

[35] Li XF (2016). Characterization of a 2016 clinical isolate of zika virus in non-human primates. EbioMedicine.

[36] Osuna CE (2016). Zika viral dynamics and shedding in rhesus and cynomolgus macaques. Nat. Med..

[37] Beck AS, Barrett AD (2015). Current status and future prospects of yellow fever vaccines. Expert. Rev. Vaccin..

[38] Guirakhoo F (2004). Safety and efficacy of chimeric yellow Fever-dengue virus tetravalent vaccine formulations in nonhuman primates. J. Virol..

[39] Dowd KA (2016). Broadly neutralizing activity of zika virus-immune sera identifies a single viral serotype. Cell Rep..

[40] Elong Ngono A (2017). Mapping and role of the CD8+T cell response during primary zika virus infection in mice. Cell Host Microbe.

[41] Manangeeswaran M, Ireland DD, Verthelyi D (2016). Zika (PRVABC59) infection is associated with T cell infiltration and neurodegeneration in CNS of immunocompetent neonatal C57Bl/6 Mice. PLoS Pathog..

[42] Guy B (2008). Cell-mediated immunity induced by chimeric tetravalent dengue vaccine in naive or flavivirus-primed subjects. Vaccine.

[43] Zhang FC, Li XF, Deng YQ, Tong YG, Qin CF (2016). Excretion of infectious Zika virus in urine. Lancet Infect. Dis..

[44] Bardina SV (2017). Enhancement of Zika virus pathogenesis by preexisting antiflavivirus immunity. Science.

[45] Li C (2016). Zika virus disrupts neural progenitor development and leads to microcephaly in mice. Cell Stem Cell.

[46] Gunther VJ (2011). A human challenge model for dengue infection reveals a possible protective role for sustained interferon gamma levels during the acute phase of illness. Vaccine.

[47] Li XF (2013). Development of chimaeric West Nile virus attenuated vaccine candidate based on the Japanese encephalitis vaccine strain SA14-14-2. J. Gen. Virol..

[48] Yuan L (2017). A single mutation in the prM protein of Zika virus contributes to fetal microcephaly. Science.

[49] Wang HJ (2014). Recombinant chimeric Japanese encephalitis virus/tick-borne encephalitis virus is attenuated and protective in mice. Vaccine.

[50] Eckels KH (1988). Japanese encephalitis virus live-attenuated vaccine, Chinese strain SA14-14-2; adaptation to primary canine kidney cell cultures and preparation of a vaccine for human use. Vaccine.

[51] Sumiyoshi H, Tignor GH, Shope RE (1995). Characterization of a highly attenuated Japanese encephalitis virus generated from molecularly cloned cDNA. J. Infect. Dis..

[52] Pardi N (2017). Zika virus protection by a single low-dose nucleoside-modified mRNA vaccination. Nature.

[53] Wen J (2017). Identification of Zika virus epitopes reveals immunodominant and protective roles for dengue virus cross-reactive CD8+T cells. Nat. Microbiol..

[54] Hirsch AJ (2017). Zika Virus infection of rhesus macaques leads to viral persistence in multiple tissues. PLoS Pathog..

[55] Quick J (2017). Multiplex PCR method for MinION and Illumina sequencing of Zika and other virus genomes directly from clinical samples. Nat. Protoc..

[56] Ornelas AM (2017). Immune activation in amniotic fluid from Zika virus-associated microcephaly. Ann. Neurol..

[57] Chen HR (2016). Dengue virus nonstructural protein 1 induces vascular leakage through macrophage migration inhibitory factor andautophagy. PLoS Negl. Trop. Dis..

[58] Chuang YC (2011). Macrophage migration inhibitory factor induced by dengue virus infection increases vascular permeability. Cytokine.

[59] Harenberg A (2016). Cytokine profile of children hospitalized with virologically-confirmed dengue during two Phase III Vaccine Efficacy Trials. PLoS Negl. Trop. Dis..

[60] Glass WG (2005). Chemokine receptor CCR5 promotes leukocyte trafficking to the brain and survival in West Nile virus infection. J. Exp. Med..

[61] Glass WG (2006). CCR5 deficiency increases risk of symptomatic West Nile virus infection. J. Exp. Med..

[62] Barba-Spaeth G (2016). Structural basis of potent Zika-dengue virus antibody cross-neutralization. Nature.

[63] Cugola FR (2016). The Brazilian zika virus strain causes birth defects in experimental models. Nature.

[64] Li SH (2013). Development and characterization of the replicon system of japanese encephalitis live vaccine virus SA14-14-2. Virol. J..

[65] Han JF (2017). Immunization with truncated envelope protein of Zika virus induces protective immune response in mice. Sci. Rep..

[66] Hamilton MA, C. RR, V. TR (1977). Trimmed Spearman-Karber method for estimating median lethal concentration in toxicity bioassays. Environ. Sci. Technol..

[67] Reed LJ, Muench H (1938). A simple method of estimating fifty percent endpoints. Am. J. Hyg..

[68] Liu Y (2017). Evolutionary enhancement of zika virus infectivity in *Aedes aegypti* mosquitoes. Nature.

[69] Dang J (2016). Zika virus depletes neural progenitors in human cerebral organoids through activation of the innate immune receptor TLR3. Cell Stem Cell.

