# Supplementary material for: Development of a chimeric Zika vaccine using a licensed live-attenuated flavivirus vaccine as backbone
Source: Nat Commun. 2018 Feb 14;9:673. doi: 10.1038/s41467-018-02975-w (PMC5813210; doi:10.1038/s41467-018-02975-w)
Supplement: Supplementary file 1 — Supplementary Information [file 41467_2018_2975_MOESM1_ESM.pdf]

Supplementary information for

Development of a chimeric Zika vaccine using a licensed live-attenuated flavivirus vaccine as backbone

* Corresponding author: qincf@bmi.ac.cn

Li et al

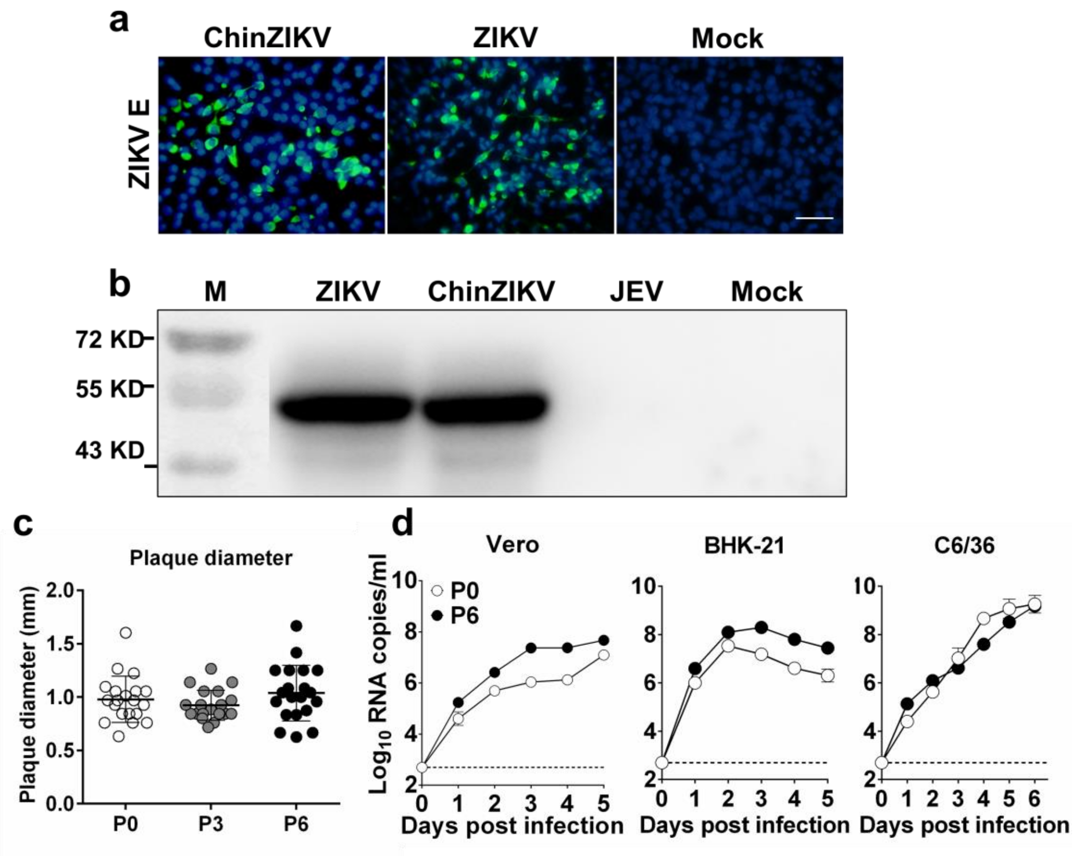

Supplementary Figure 1. Construction and characterization of ChinZIKV. (**a**) Immunostaining of ChinZIKV-, ZIKV-, or mock-infected BHK-21 cells at 48 h post-infection with a commercial monoclonal antibody against the ZIKV E protein. Scale bar: 100 μm. (**b**) Western blotting of C6/36 cell lysates infected with ZIKV, ChinZIKV, JEV or mock infected, 48 h post-infection with a commercial monoclonal antibody against the ZIKV E protein. (**c**) The average plaque diameter was calculated from 20 plaques for the P0, P3 and P6 viruses. (**d**) Growth curves of the recovered ChinZIKV and the passaged virus (P6) in Vero, BHK-21 and C6/36 cells using an MOI of 0.01. The data are representative of at least three independent experiments, and error bars indicate the SD.

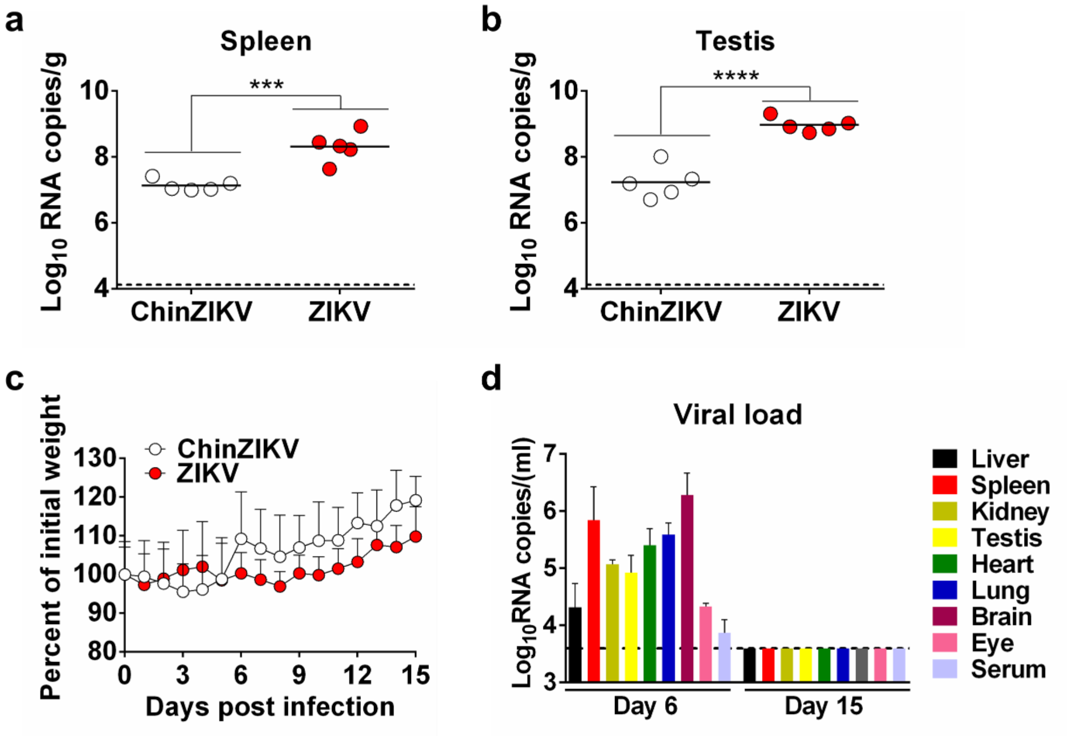

Supplementary Figure 2. Tissue distribution of ChinZIKV or ZIKV in A129 mice. Four-week-old male A129 mice were infected with 10^3 PFU of the indicated viruses by the s.c. route. Viral RNA loads in spleens (**a**) and testis (**b**) at day 3 post-infection were determined by RT-qPCR. (**c-d**) Six-week-old male A129 mice (n=4) were infected s.c. with 10^3 PFU of ChinZIKV. The body weight of infected mice was monitored for 15 days (**c**); Selected organs were collected at days 6 and 15 post infection for viral RNA detection (**d**). Dotted lines indicate the detection limit. The data are representative of at least three independent experiments, and error bars indicate the SD. Significance was calculated using a Student's t test (***, *P-value*<0.001; ****, *P-value*<0.0001).

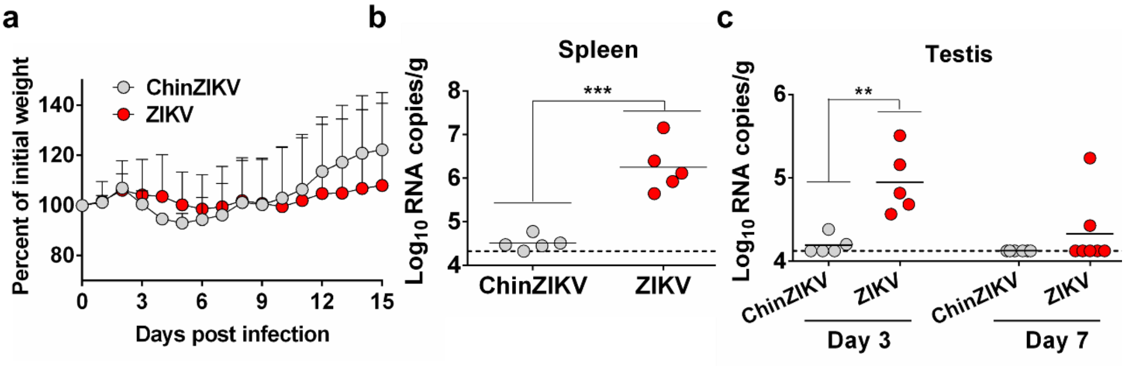

Supplementary Figure 3. Tissue distribution of ChinZIKV or ZIKV in BALB/c mice. Four-week-old male BALB/c mice were infected with 10^5 PFU of the indicated viruses by the i.p. route. The body weight of the infected mice (n=5) was monitored for 15 days (**a**). Viral RNA loads in spleens at day 3 post-infection (**b**) and testis, at days 3 and 7 post-infection (**c**) were determined by RT-qPCR. Dotted lines indicate the detection limit. The data are representative of at least three independent experiments, and error bars indicate the SD. Significance was calculated using a Student's t test (**, *P-value*<0.01; ***, *P-value*<0.001).

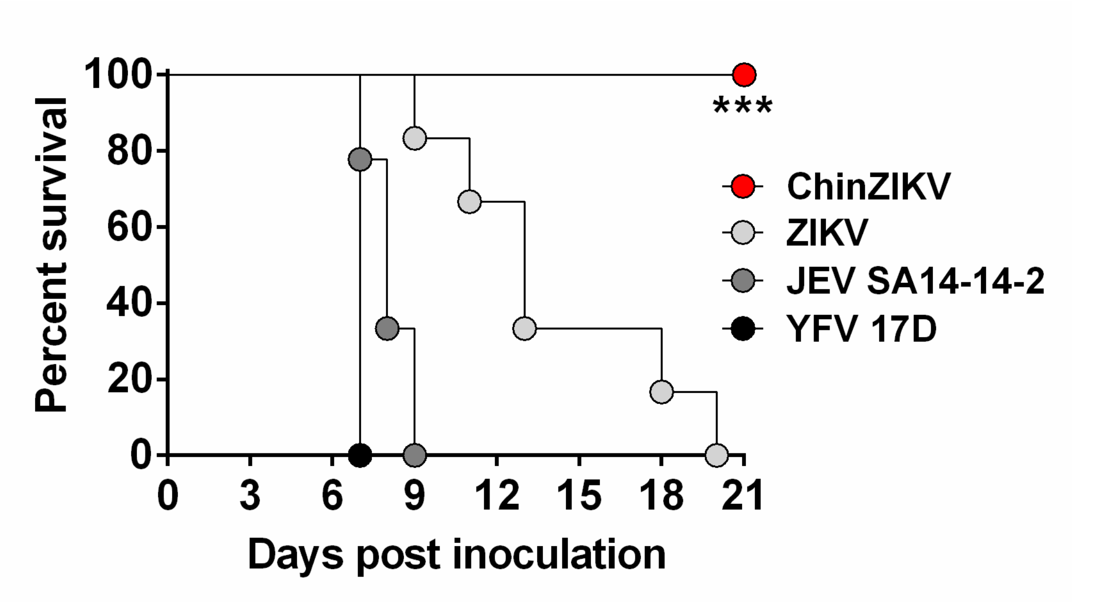

Supplementary Figure 4. Neurovirulence of ChinZIKV and related flaviviruses in mice. One-day-old suckling BALB/c mice were inoculated i.c. with 10 PFU of ChinZIKV (n=7), ZIKV (n=6), JEV SA14-14-2 (n=9) or YFV 17D (n=7). Animals were monitored for 21 days after inoculation. Kaplan-Meier survival curves were analyzed by a log rank test (***, *P-value*<0.001).

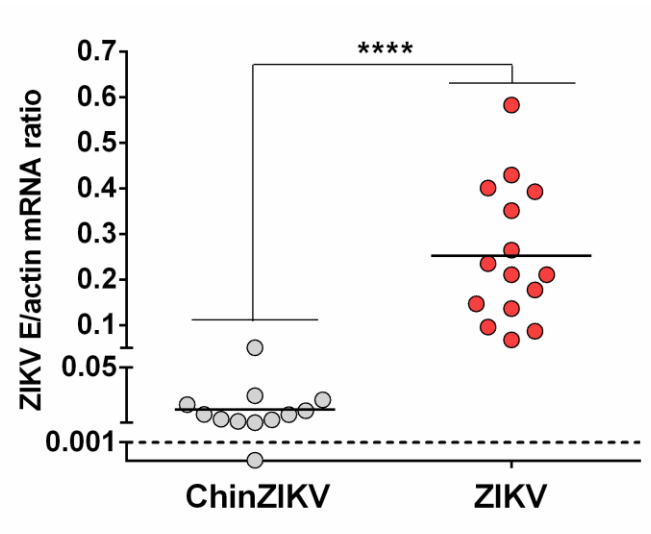

Supplementary Figure 5. Comparison of the infectivity of ChinZIKV and ZIKV in *Aedes aegypti*. Female *Aedes aegypti* mosquitoes were anaesthetized on a cold tray, and subsequently microinjected with 0.2 PFU of ZIKV or ChinZIKV into the thorax. Viral RNA loads at day 6 post-inoculation were determined by RT-qPCR. Each dot represents a mosquito. The dotted line indicates the detection limit. The data are representative of at least three independent experiments, and error bars indicate the SD. Significance was calculated using a Student's t test (****, *P-value*<0.0001).

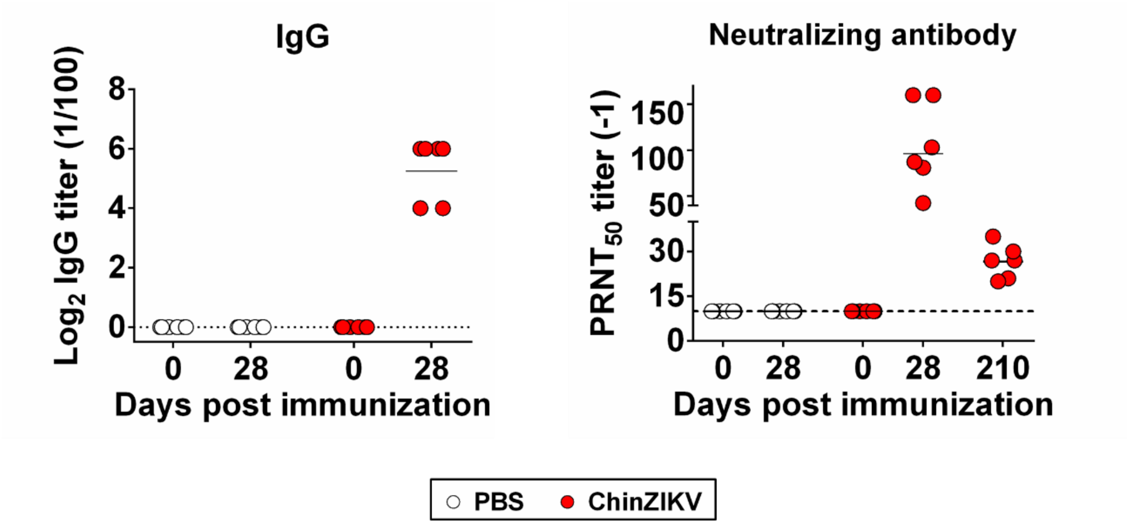

Supplementary Figure 6. Immunogenicity of ChinZIKV in BALB/c mice. Four-week-old female BALB/c mice were immunized s.c. with 10^4 PFU of ChinZIKV (n=6) or PBS as a control (n=6). Sera from the immunized mice were collected at days 0, 28 and 210 post-immunization. ZIKV-specific IgG and neutralizing antibody titers were determined by ELISA and $PRNT_{50}$, respectively. Dotted lines indicate the detection limits of detection.

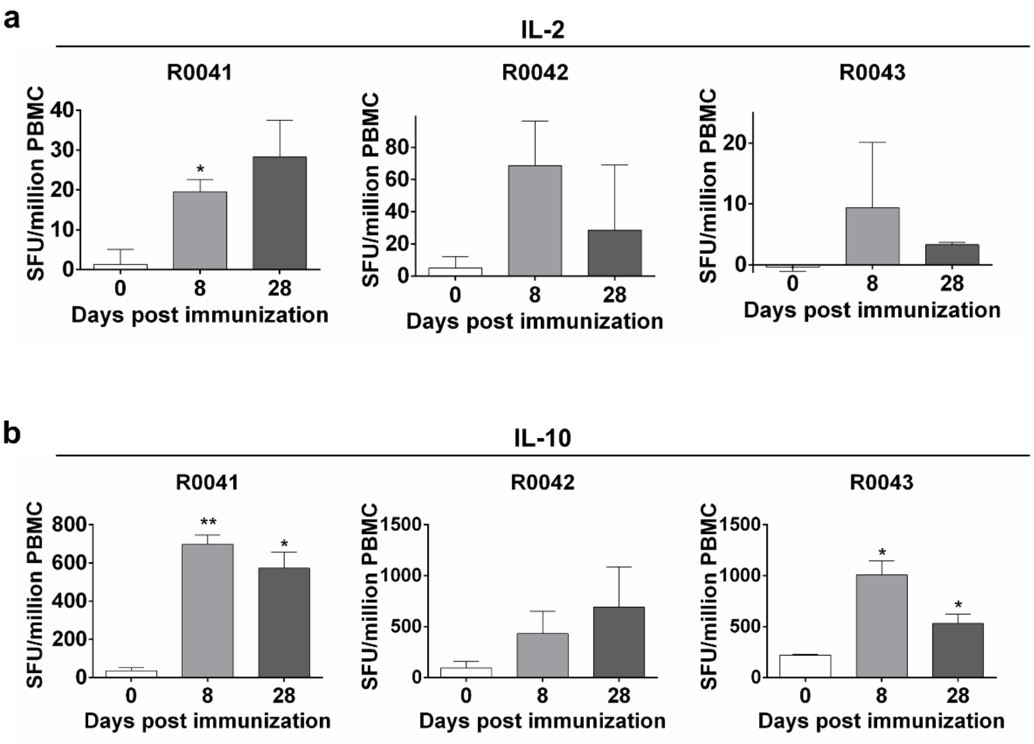

Supplementary Figure 7. Cellular immune responses in monkeys immunized with ChinZIKV. PBMCs of immunized monkeys were collected at the indicated times. The production of IL-2 (**a**) and IL-10 (**b**) by PBMCs, in response to stimulation with the ZIKV E protein, were measured by ELISPOT assay and expressed as spot-forming units (SFU) per 10^6 PBMCs. Experiments were performed in duplicate (error bars represent SD). Statistical significance between pre- and post-immunization conditions was calculated using a one-way ANOVA with multiple comparison tests (*, *P-value*<0.05; **, *P-value*<0.01).

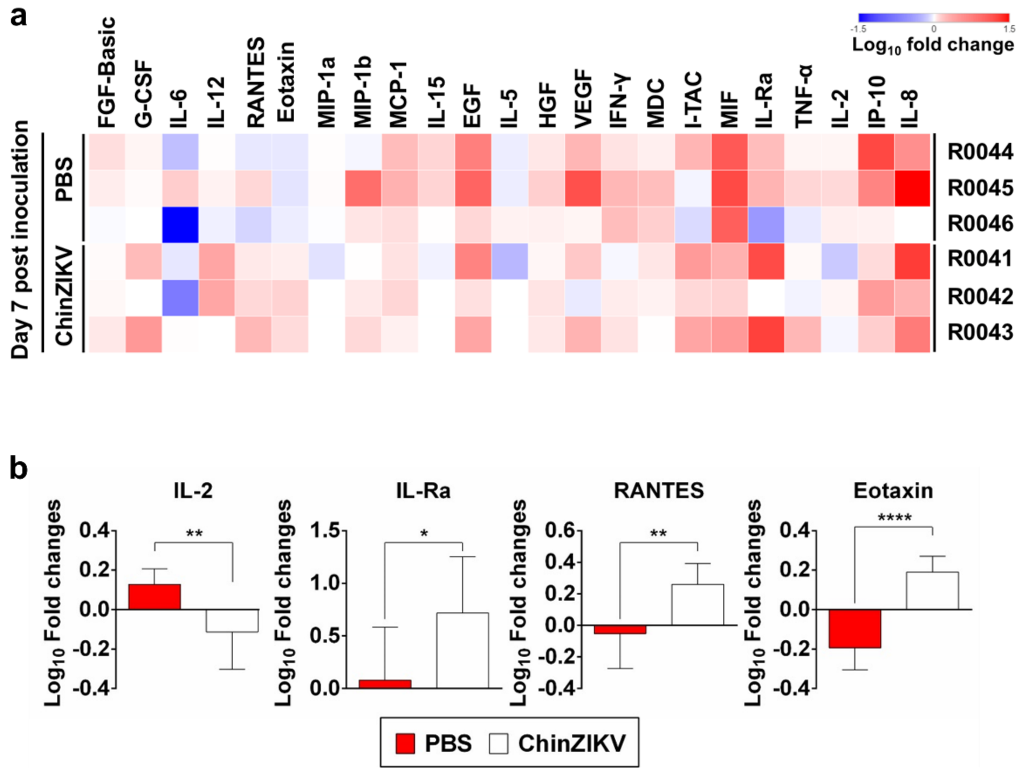

Supplementary Figure 8. Cytokine production profile in ChinZIKV-immunized monkeys following ZIKV challenge. (**a**) Heatmap of cytokine levels in ChinZIKV- or PBS-immunized monkeys following ZIKV challenge. The levels of various cytokines in the sera of immunized monkeys on day 7 post-challenge were measured using a Monkey Cytokine Magnetic 29-Plex Panel kit. Each cytokine level is summarized as the log10 of the ratio relative to baseline (Day 0 post challenge). (**b**) Cytokine production in the sera of immunized monkeys at day 7 post-challenge determined by a Monkey Cytokine Magnetic 29-Plex Panel kit. Experiments were performed in duplicate (error bars represent SD). Significance was calculated using a Student's t test (*, *P-value*<0.05; **, *P-value*<0.01; ****, *P-value*<0.0001).

Supplementary Table 1. Nucleotide changes present in ChinZIKV P6.

Nucleotide position	Nucleotide change	Amino acid position	Amino acid change
721	A→G	prM-82	His-Arg
2706	A→C	NS1-72	Silent
2858	C→T	NS1-122	Silent
3366	A→G	NS1-292	Ser-Gly
3614	A→G	NS1-374	Silent
8186	A→T	NS5-165	Silent

Supplementary Table 2. Oligonucleotides and fluorogenic probes used in this study.

Name	Sequence (5′-3′)	Purpose
ZIKV-prME-F	CATAGCTTGTGCAGGCGCCGTGGA GGTCACTAGACGTGGGAATGCAT	OL-PCR
ZIKV-prME-R	CAGTGTCAGCATGCACGGCTGTGG ATAAGAAGATCAACACTCCCCCTA	OL-PCR
JEV-NS1-F	TAGGGGGAGTGTTGATCTTCTTATC CACAGCCGTGCATGCTGACACTG	OL-PCR
BspE1	CCATTTTCTGTCCGGAATCGT	OL-PCR
ZIKV-1086F	CCGCTGCCCAACACAAG	qRT-PCR
ZIKV-1162R	CCACTAACGTTCTTTTGCAGACAT	qRT-PCR
ZIKV1107-probe	(FAM)-AGCCTACCTTGACAAGCAATCAGACACTCAA-(BHQ1)	qRT-PCR
A. aegypti actin-F	GAACACCCAGTCCTGCTGACA	qRT-PCR
A. aegypti actin-R	TGCGTCATCTTCTCACGGTTAG	qRT-PCR
A. aegypti actin-probe	(FAM)-AGGCCCCGCTCAACCCGAAG-(TAMRA)	qRT-PCR

Supplementary Table 3. Basic information about rhesus macaques used in this study.

Inoculation	ID	Gender	Weight (kg)	DOB#
ChinZIKV	R0041*	F	6.0	08/01/11
	R0042*	F	5.3	01/02/11
	R0043*	F	5.3	05/09/11
	R3092	F	5.1	02/16/11
	R4020	F	5.8	05/02/11
ZIKV	R0082	F	5.9	02/03/11
	R3076	F	5.9	03/05/11
	R3016	M	5.0	03/12/11
PBS	R0044*	F	5.0	08/05/11
	R0045*	F	4.5	07/10/12
	R0046*	F	5.1	02/11/11

#, DOB, date of birth.
*, Challenged with 10^3 PFU of ZIKV.

Supplementary Table 4. Original data for cytokine heatmap in ChinZIKV- ZIKV-inoculated monkeys.

Inoculation	ID	Dose (PFU)	Cytokine production*																			
			FGF-Basic	IL-1b	IL-12	RANTES	Eotaxin	GM-CSF	MIP-1b	MCP-1	IL-15	EGF	HGF	IFN-r	I-TAC	MIF	IL-Ra	TNF-a	IL-2	IP-10	MIG	IL-8
ZIKV	R0082	10^5	0.374	0.976	0.448	0.256	0.153	-0.041	0.556	0.602	0.643	0.697	0.483	0.636	-0.530	1.364	0.233	0.975	-0.014	0.240	0.436	2.252
	R3076	10^5	0.159	0.225	0.403	0.164	0.065	-0.060	0.356	0.560	0.370	0.747	0.223	0.393	-0.402	1.393	0.025	0.518	0.219	-0.011	0.109	2.072
	R0016	10^5	0.161	0.230	0.354	-0.555	0.234	0.517	0.167	0.744	0.519	0.749	0.173	0.438	-0.147	1.522	0.315	0.000	0.425	0.110	0.280	1.650
ChinZIKV	R0041	10^5	0.009	0.130	-0.015	-0.069	-0.383	-0.237	0.046	0.204	-0.014	0.162	0.017	0.029	-0.179	0.595	0.296	-0.367	-0.260	0.046	-0.052	1.144
	R0042	10^5	0.046	0.323	0.381	-0.070	0.153	-0.018	0.211	0.465	0.241	0.241	0.203	0.252	0.033	0.877	0.337	-0.180	0.000	0.154	0.412	1.314
	R0043	10^5	0.184	0.155	0.030	0.216	-0.302	-0.024	0.509	0.350	0.021	0.418	0.157	0.274	-0.619	1.054	-0.018	0.327	0.168	-0.194	-0.184	1.236

*, Each cytokine level on day 7 post-immunization is summarized as the log_{10} of the ratio relative to baseline (day 0 p.i.)

Supplementary Table 5. Original data for cytokine heatmap in ChinZIKV-immunized monkeys following ZIKV challenge.

ID	Dose (PFU)[#]	Cytokine production*																			
		FGF-Basic	G-CSF	IL-6	IL-12	RANTES	Eotaxin	MIP-1a	MIP-1b	MCP-1	IL-15	EGF	IL-5	HGF	VEGF	IFN-r	MDC	I-TAC	MIF	IL-Ra	TNF-a
R0044	10^3	0.192	0.064	-0.371	0.020	-0.137	-0.133	0.019	-0.054	0.395	0.249	0.745	-0.106	0.142	0.432	0.164	0.093	0.435	0.966	0.388	0.065
R0045	10^3	0.106	0.028	0.296	0.076	0.234	-0.157	0.024	0.848	0.458	0.252	0.899	-0.106	0.284	1.016	0.425	0.385	-0.065	1.051	0.442	0.240
R0046	10^3	-0.032	0.005	-1.654	-0.089	-0.246	-0.104	-0.017	0.152	0.196	0.013	0.238	0.076	0.087	0.065	0.387	0.280	-0.219	0.927	-0.588	-0.125
R0041	10^3	0.029	0.402	-0.139	0.531	0.130	0.101	-0.169	0.012	0.170	-0.074	0.722	-0.411	0.054	0.322	-0.046	0.147	0.590	0.457	1.049	0.044
R0042	10^3	0.048	0.000	-0.757	0.524	0.231	0.263	0.000	0.128	0.240	-0.008	0.181	0.000	0.158	-0.121	0.110	0.078	0.231	0.257	0.000	-0.072
R0043	10^3	0.137	0.599	0.020	0.000	0.420	0.210	0.000	0.225	0.089	0.000	0.530	0.000	0.136	0.450	0.164	0.010	0.526	0.595	1.101	0.421

*, Each cytokine level on day 7 post-challenge is summarized as the $\log_{10}$ of the ratio relative to baseline (day 0 p.i.)
#, Immunized monkeys were challenged s.c. with 10^3 PFU of ZIKV on day 55 post-immunization.
